# Cognitive Functioning in Adolescents with Self-Reported ADHD and Depression: Results from a Population-Based Study

**DOI:** 10.1007/s10802-016-0160-x

**Published:** 2016-05-03

**Authors:** Arunima Roy, Albertine J. Oldehinkel, Catharina A. Hartman

**Affiliations:** 1 Interdisciplinary Centre Psychopathology and Emotion Regulation (ICPE), University of Groningen, University Medical Centre Groningen, CC 72, P.O. Box 30.001, 9700 RB Groningen, Netherlands; 2 Division of Child Psychiatry, McGill University, Montreal Children’s Hospital, Montreal, Quebec Canada

**Keywords:** ADHD, Depression, Cognition, Adolescents

## Abstract

This study aims to assess cognitive functioning differences among adolescents with retrospectively self-reported: ADHD and an onset of depression, only ADHD, only depression, and neither ADHD nor depression. Data from the Tracking Adolescents’ Individual Lives Survey (TRAILS) cohort was used in this study. Neuropsychological functioning was assessed in 1549 adolescents, at baseline and follow-up (mean ages 11 and 19 years). The Composite International Diagnostic Interview was used to classify adolescents into 4 groups: ADHD with onset of depression, only ADHD, only depression, and neither ADHD nor depression. Linear mixed effects models were used to analyse group differences in cognitive functioning at baseline and follow-up, and the change in cognitive functioning between these 2 time-points. Results showed a significant main effect of group on response time variability at baseline, working memory maintenance at follow up, and change in response time variability scores between baseline and follow-up. As compared to the healthy and depressed-only groups, adolescents with only ADHD showed longer response time variability at baseline and, which declined between baseline and follow-up. Adolescents with ADHD plus depression showed higher reaction time for working memory maintenance than the depressed only and healthy groups at follow-up. In conclusion, adolescents with self-reported ADHD show poorer cognitive functioning than healthy adolescents and those with only depression. Amongst adolescents with ADHD, specific cognitive domains show poor functioning depending on the presence or absence of comorbid depression. While adolescents with only ADHD have lower reaction time variability, those with comorbid depression have poorer working memory maintenance.

**Electronic supplementary material:**

The online version of this article (doi:10.1007/s10802-016-0160-x) contains supplementary material, which is available to authorized users.

Attention Deficit Hyperactivity Disorder or ADHD is a neurodevelopmental disorder of childhood that often persists into adolescence and adulthood (DSM-5 American Psychiatric Association 2013). Apart from symptoms of hyperactivity, impulsivity and inattentiveness, affected individuals show a wide range of cognitive functioning deficits, such as problems in planning, working memory, inhibition and attention (Cortese et al. 2015; Sebastian et al. 2014; Willcutt et al. 2005). Cognitive dysfunction in ADHD is heterogeneous; not only are cognitive deficits absent in many cases of ADHD, affected cognitive domains also vary widely across individuals with ADHD (Martel et al. 2011). There is, thus, poor consensus regarding the specific cognitive profile associated with ADHD (Nigg et al. 2005; Sergeant et al. 2003; Sergeant et al. 2002; Sjowall et al. 2013; Sonuga-Barke 2003). A suspected source of this variability may be the high comorbidity of ADHD with other psychiatric disorders (Pauli-Pott et al. 2014). Comorbidities may increase the severity of existing cognitive deficits in children and adolescents with ADHD (Crawford et al. 2006). In addition, comorbid disorders may modify the cognitive functioning profile associated with ADHD. Although not much is known yet about the deficits associated with ADHD in combination with specific comorbid disorders, it is likely that cognitive profiles differ for each of these conditions (Larochette et al. 2011; Pauli-Pott et al. 2014; Vloet et al. 2010; Willcutt et al. 2001). In this study we will focus on the cognitive functioning and development of depression in adolescents with and without ADHD.

Depression develops in 30 to 70 % of individuals with ADHD (Chronis-Tuscano et al. 2010; Fergusson et al. 2010; Jensen et al. 1993; Meinzer et al. 2014) Results from these studies show that both boys and girls with ADHD, across a wide age range from 4 to 18 years, are likely to develop depression. Comorbid depression leads to further impairments in prognosis and quality of life (Angold et al. 1999; Bagwell et al. 2001; Biederman et al. 1993; Blackman et al. 2005; Coleman 2008). A knowledge of the cognitive profile associated with ADHD and comorbid depression may assist in understanding the ADHD-depression relationship better. Not many studies though, exist on the cognitive functioning of individuals with ADHD and depression. A recent meta-analysis indicated that depression is weakly to moderately associated with deficits in various cognitive domains (Snyder 2013), several of which are also common to ADHD (Nigg 2005).

The combined condition of ADHD and depression may give rise to a unique cognitive profile that is distinguishable from that of either disorder alone (either ADHD or depression) in the following manners. First, as found in previous studies, comorbidities may increase the severity of cognitive deficits (Crawford et al. 2006). Therefore, individuals with ADHD plus depression may have a worse cognitive functioning than those with either ADHD or depression. Second, cognitive deficits may be a vulnerability factor, facilitating the onset of depression (Austin et al. 2001). Thus, in adolescents with ADHD too, cognitive dysfunction may precede an onset of depression. Prospective studies on long-term outcomes of cognitive (dys)function in ADHD are limited and consequently this idea has not been explored yet. Third, cognitive maturation is often delayed amongst individuals with ADHD (Rajendran et al. 2013; Shaw et al. 2006, 2007). A development of depression during this period may further interfere with the process of cognitive maturation and thereby differentiate cases of ADHD plus depression from cases with either ADHD or depression.

This study aims to understand if ADHD with an onset of depression is associated with a unique cognitive profile. In a large population-based sample with retrospective self-reports of ADHD and depression, we assess cognitive functioning differences at two time-points among adolescents with ADHD plus depression, only ADHD, and controls with or without depression. Further, we also assess group differences in the change in cognitive functioning between the two-time points.

## Methods

### Cohort

The data were collected as part of the TRacking Adolescents’ Individual Lives Survey (TRAILS), an ongoing Dutch prospective cohort study on psychosocial development and mental health of adolescents. TRAILS involves bi- or triennial measurements from ages 11 onwards and consists of two separate cohorts: one population-based and another clinic-based. (de Winter et al. 2005; Huisman et al. 2008; Oldehinkel et al. 2015b; Ormel et al. 2012). This study is based on data from the TRAILS population cohort.

Children were recruited from five municipalities in the north of The Netherlands, including both urban and rural areas. Primary school participation was requisite for inclusion. Of the 2935 children who met these criteria, 2230 (76.0 %) provided informed consent from both parent and child to participate in the study. The present study utilises data from the first and fourth wave. The first wave (T1) ran from March 2001 to July 2002, the fourth (T4) from October 2008 to September 2010 (T4). Mean age at T1 was 11.1 years (SD = 0.56), and 50.8 % were girls. Response rate at T4 was 83.4 % (*N* = 1881, mean age = 19.1, *SD* = 0.60, 52.3 % girls), of whom 84.2 % (*N* = 1584) completed the below-described diagnostic interview. (For an overview of the mean age and gender distributions of participants please see supplementary material, S1). To ensure all onsets of depression were between T1 and T4, we excluded participants who developed a depression prior to T1 (*n* = 35). This gave a final sample size of 1549 adolescents. T1 is referred to as baseline and T4 as follow-up in following sections of the paper.

The Amsterdam Neuropsychological Tasks used in this study to assess cognitive function was also part of two previous TRAILS publications on depressive problems in adolescents, but not specific to ADHD (Nederhof et al. 2014; Oldehinkel et al. 2015a). These studies used different ANT parameters than here focusing on either attention style (Nederhof et al. 2014) or emotion recognition (Oldehinkel et al. 2015a) in relation to depressive symptoms (rather than disorder). Further, data from the TRAILS cohort has been used in two studies to study mediators (other than cognitive function) of the ADHD-depression association (Roy et al. 2015; Roy et al. 2014). Thus, the content of the current manuscript does not overlap with these previous publications.

### Measures

Cognitive functions were assessed at baseline and follow-up using the Amsterdam Neuropsychological Tasks program (ANT) (please see supplementary material S2 for further details). The ANT has proven to be a sensitive and valid tool in assessing cognitive functions (de Sonneville et al. 1993), especially for population and clinic based samples of attention-deficit/hyperactivity disorder (Hanisch et al. 2004; Slaats-Willemse et al. 2003). Previously, studies have used the ANT to assess sustained attention, processing speed (Huijbregts et al. 2002), focused attention, working memory, cognitive flexibility (Lazeron et al. 2006) and response inhibition (Groot et al. 2004). A detailed description of the ANT is available in previous studies (Nederhof et al. 2014; van Deurzen et al. 2012). In short, the ANT is a computer-aided test to assess cognitive capacities, with high sensitivity and validity. We used five subtasks from the ANT: (a) Baseline Speed task, (b) Pattern Recognition task, (c) Sustained Attention - dots task, (d) Memory Search - letters task, and (e) Shifting Attentional Set - visual task. All children were tested individually by trained undergraduate psychology students. Use of prescribed medications (if any) was not withheld prior to the assessments. Verbal instructions, emphasizing both speed and accuracy of performance, and practice trials preceded each task. Six output measures were calculated from the above tasks: processing speed (from Baseline Speed), focussed attention (from Pattern Recognition), response time variability (from Sustained Attention Dots), working memory maintenance (from Memory Search - letters), response inhibition (from Shifting Attentional Set – visual), and cognitive flexibility (from Shifting Attentional Set – visual). For all output measures, except response time variability, mean Reaction Time (RT) in milliseconds was defined as the outcome parameter (for response time variability, within-subject variability in RT of the sustained attention – dots task was defined as the outcome). Only RTs to correct trials were used in the analyses. The Baseline Speed task required responses from both the dominant and the non-dominant hand. The outcome parameter for this task was defined as mean RT of responses from both hands. For all outcomes, RTs with an absolute z-score greater than or equal to 4 were defined as missing (Stevens 2009). Correlations between the measures were generally weak (mean *r* = 0.24, range = 0.07–0.55), suggesting limited overlap.

Psychiatric disorders were assessed at follow-up by means of the World Health Organization Composite International Diagnostic Interview (CIDI), version 3.0. The CIDI is a structured diagnostic interview that yields lifetime and current diagnoses according to the definitions and criteria of the Diagnostic and Statistical Manual of Mental Disorders (DSM-IV). The CIDI has been used in a large number of surveys worldwide, and shown to have good concordance with clinical diagnoses (Haro et al. 2006; Kessler et al. 2004, 2009). In addition to the occurrence of psychiatric disorders, the CIDI yields their age at onset and age at last occurrence. The CIDI has good reliability and validity for most diagnoses (Wittchen 1994). In this study, information on CIDI diagnoses of ADHD and depression were used. The ADHD screening questions were operationalised as: (1) a history of concentration problems (such as quickly losing interest in work and games, inability to concentrate on and finish work, not listening to other people when spoken to) prior to the age of 7 that lasted a minimum of 6 months and seemed excessive compared to peers, and/or (2) a history of hyperactivity-impulsivity (such as fidgeting, restlessness and impatience) present before the age of 7 that lasted a minimum of 6 months. A positive response to either of these two questions was followed up by a full DSM-IV based assessment of ADHD. This includes the presence of the requisite number of ADHD symptoms as defined in DSM-IV, an onset before the age of 7, and the presence of functioning impairments. Depression was operationalized as a lifetime diagnosis of Major Depressive Episode (with or without (hypo)manic symptoms), Dysthymia, or Minor Depressive Disorder. Validity of the CIDI data was supported by prospective parent-, self-, and teacher-reports as assessed with the Child Behaviour Checklist (CBCL), Youth Self Report (YSR), Adult Self Report (ASR), and Teacher’s Checklist of Pathology (TCP) from the first wave onwards (supplementary material S3, Tables III–IV) (Achenbach 1991a, b). The TCP, developed by the TRAILS team (de Winter et al. 2005), is based on items from the Teacher’s Report Form (Achenbach and Rescorla 2001). As several participants in the TRAILS cohort were from the same class, the TCP was developed to ease the filling up of questionnaires by teachers. Teacher-reported ADHD scores in this study combined the attention and hyperactivity/impulsivity problem items of the TCP (corresponding to the CBCL-YSR attention problems scales).

Information on medication use between baseline and follow-up was collected using parent and self-report questionnaires. Use of anti-depressants included imipramine, clomipramine, amitriptyline, nortriptyline, fluoxetine, citalopram, paroxetine, sertraline, fluvoxamine, escitalopram, moclobemide, venlafaxine; while medications for ADHD involved methylphenidate, atomoxetine, and dexamphetamine.

### Analyses

Based on lifetime CIDI diagnoses at follow-up, participants were categorized into four groups: (1) ADHD with an onset of depression (Group A+D), (2) only ADHD (Group A), (3) only an onset of depression (Group D), and (4) comparison: neither ADHD nor an onset of depression (Group C). Mean self-reported ADHD and depressive symptom scores (assessed using the YSR at T1, T2, T3 and the ASR at T4) at the four assessment waves were plotted to visualise the approximate time-point of divergence in symptom scores amongst the four groups.

Linear mixed effects models were used to analyse differences in cognitive functioning among these groups at baseline and follow-up as well as change in cognitive functioning between baseline and follow-up (slope). We adjusted for the effects of age at baseline, gender, and medication use in the analyses by including these variables as covariates. The (co)variance matrix was set to unstructured (i.e., freely estimated) for all analyses. For the cognitive outcome measures that showed group differences, we additionally plotted the standardized RTs per group at baseline and follow-up and estimated the effect sizes of comparisons with group C using Cohen’s *d*.

A sensitivity analysis was conducted as a second step to determine if (lack of) differences in cognitive functioning among groups were due to the presence of psychiatric disorders other than ADHD or depression in the comparison group. For this, a new comparison group (Group H: healthy) was formed by excluding participants with CIDI diagnoses of other psychiatric disorders from group C, and the above-described linear mixed effects analyses were re-run to test cognitive functioning differences among groups A+D, A, D and H. Age at baseline, gender and medications were included as covariates.

A second sensitivity analysis was additionally performed to explore the validity of the CIDI diagnoses. Data on parent-reported ADHD as assessed by the Diagnostic Interview Schedule for Children (DISC) were available for the parallel TRAILS Clinical (high-risk) Cohort at baseline. A comparison of the DISC with the CIDI outcomes showed that almost 80 % of children with an ADHD diagnosis at baseline according to the DISC did not have a CIDI diagnosis of ADHD in adulthood (note that both interviews required an onset of ADHD before age 7). Conversely, 80 % of those who had a CIDI diagnosis of ADHD also had a DISC diagnosis of ADHD in childhood. These results suggest the presence of relatively few false positives in our current sample (with the use of CIDI), yet many false negatives. To assess the possibility of false negative ADHD diagnoses using the CIDI, we reformed the comparison group by excluding participants with a high baseline CBCL or TCP ADHD symptom score (i.e., scores higher than 1.5 *SD* in the total sample).

Analyses were performed using SPSS v. 22.0.0 (IBM Corp., Armonk, NY) and graphs were plotted using MATLAB 2012b (The MathWorks, Inc. Natick, MA). All tests were two-tailed and a *p* ≤ 0.05 was considered statistically significant. For the post-hoc pairwise tests, results were adjusted using the Benjamini-Hochberg False Discovery Rate (FDR) (Benjamini and Hochberg 1995) and the threshold for statistical significance was set at *p* ≤ 0.05.

## Results

Of the 1549 adolescents in our study, and based on the CIDI, 3.6 % received a diagnosis of ADHD (*n* = 56), and 19.6 % a diagnosis of depression (*n* = 303). Amongst those with ADHD, 37.5 % developed depression between baseline and follow-up (*n* = 21). These numbers are consistent with estimates of lifetime mental health problems obtained in late adolescence (Merikangas et al. 2010). Of the total sample, 37.5 % of adolescents with ADHD and 5.6 % of adolescents with depression received medication at some point between the four assessment waves. Participants with and without medications did not differ in their cognitive functioning scores at baseline or follow-up (for further details please see supplementary material S3 [Tables I–II]).

Parent- (CBCL) and teacher- (TCP) reports, available at baseline, were used to estimate the number of adolescents in the A+D and A groups with ADHD scores >1.5 SD. Parent-reports showed > 1.5 *SD* scores in 5 participants from group A+D (*n* = 21) and 12 participants from group A (*n* = 35). Using teacher-reports, these numbers were five in group A+D and seven in group A. Scores greater than 1.5 *SD* on either parent or teacher reports were found among 10 participants from group A+D and 13 participants from group A. Mean parent-reported ADHD symptom scores in group A+D and A were 0.87 (*SD* = 0.58) and 1.01 (*SD* = 0.45), respectively. Mean teacher-reported ADHD symptom scores were 1.41 (*SD* = 1.20) and 1.16 (*SD* = 1.18) for groups A+D and A, respectively.

**Fig. 1:**
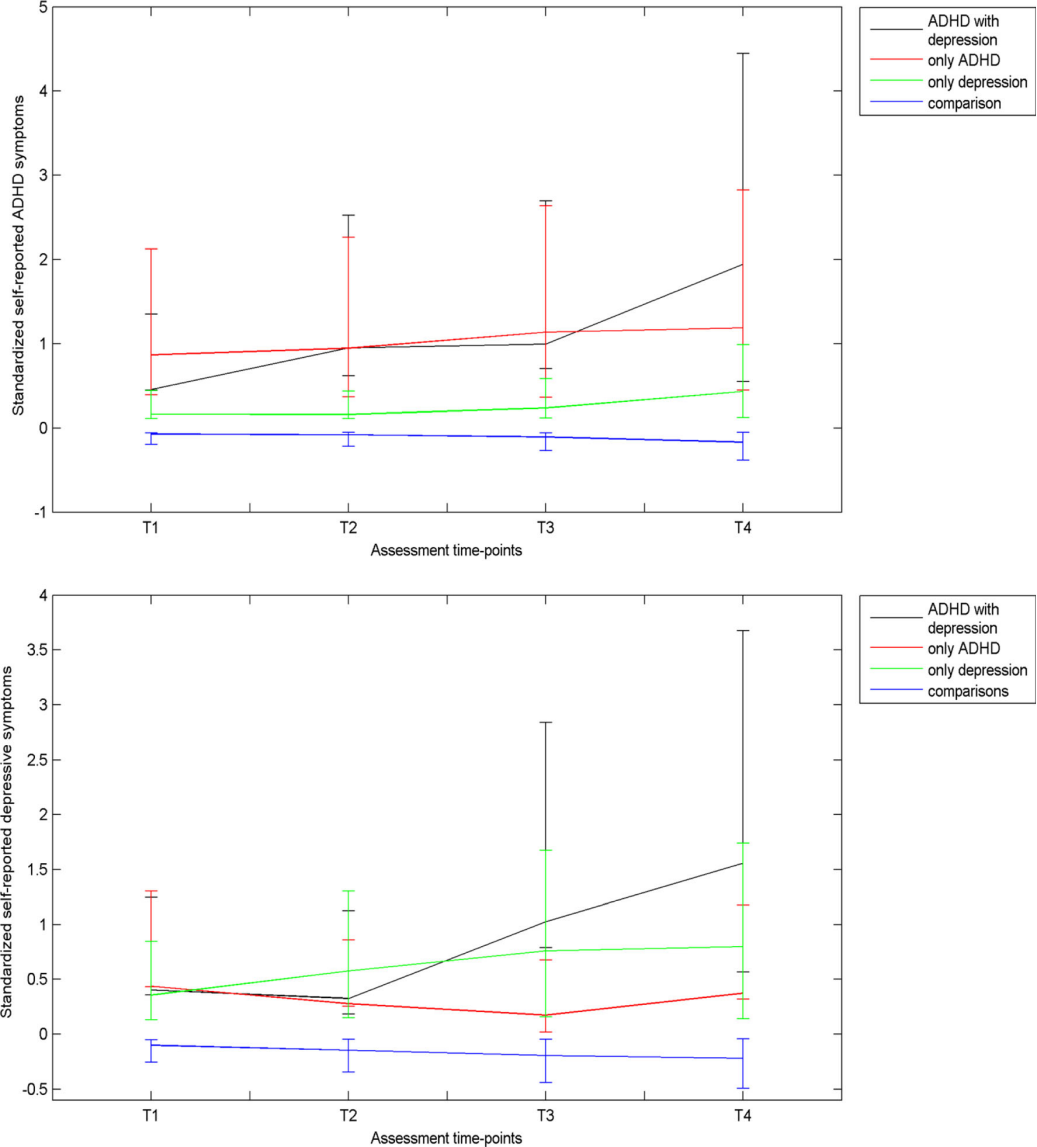
Mean ADHD and depressive symptom scores (assessed using Youth Self Reports at T1, T2, T3 and Adult Self Reports at T4) between ages 11 and 19 years for all four groups

Figure 1 presents mean self-reported ADHD and depressive symptom scores assessed (using the YSR and ASR) at each time point for the four groups. At T1, group A+D had higher self-reported ADHD symptoms than group C (*d* = 0.53, *p* = .019), while group A had higher ADHD symptoms than groups D (*d* = 0.67, *p* < .001) and C (*d* = 0.88, *p* < .001). ADHD symptom scores at T4 of group A+D was higher than groups A (*d* = 0.66, *p* = 0.004), D (*d* = 1.41, *p* < .001) and C (*d* = 2.05, *p* < .001), and of group A was higher than groups D (*d* = 0.66, *p* < .001) and C (*d* = 1.24, *p* < .001). Depressive symptoms at T1 were higher amongst groups A+D (*d* = 0.53, *p* = .023), A (*d* = 0.48, *p* = .002) and D (*d* = 0.43, *p* < .001) than group C. At T4, mean depressive scores were high for group A+D as compared to group A (*d* = 0.98, *p* < .001), group D (*d* = 0.64, *p* < .001) and group C (*d* = 1.73, *p* < .001). For group D at T4, depressive symptoms were higher than group A (*d* = 0.35, *p* = .010) and group C (*d* = 1.01, *p* < .001).

Table 1 presents mean age at onsets of ADHD and depression, and mean RTs for cognitive outcome measures in the five groups A+D, A, D, C, and H. Pearson’s correlations revealed statistically significant relationships between RTs at baseline and follow-up for all cognitive outcome measures (processing speed: *r* = 0.44; focussed attention: *r* = 0.37; response time variability: *r* = 0.50; working memory maintenance: *r* = 0.46; inhibition: *r* = 0.34; cognitive flexibility: *r* = 0.34; all *p* < .001). For further details on the association between baseline and follow-up cognitive functions in the TRAILS sample, please see Boelema et al. (2013).

**Table 1:** Summary of group characteristics, including age at onset of ADHD and depression, and reaction time for cognitive measures at baseline and follow-up

	Groups^*^				
	A+D *N* = 21, 52 % girls (mean ± SD)	A *N* = 35, 37 % girls (mean ± SD)	D *N* = 282, 73 % girls (mean ± SD)	C *N* = 1211, 50 % girls (mean ± SD)	H *N* = 888, 49 % girls (mean ± SD)
Age at onset of ADHD^**^	6 ± 2	5 ± 2	–	–	–
Age at onset of depression^**^	14 ± 2.4	–	15 ± 2.2	–	–
Processing speed^#^					
Baseline	328 ± 38	345 ± 42	329 ± 38	327 ± 39	325 ± 37
Follow-up	243 ± 24	256 ± 25	252 ± 23	250 ± 25	250 ± 24
Focused attention^#^					
Baseline	1467 ± 604	1533 ± 356	1462 ± 469	1420 ± 443	1404 ± 442
Follow-up	795 ± 301	787 ± 238	810 ± 259	808 ± 258	799 ± 252
Response time variability^#^					
Baseline	1898 ± 956	2095 ± 762	1688 ± 828	1652 ± 810	1617 ± 806
Follow-up	974 ± 391	916 ± 365	862 ± 366	858 ± 366	849 ± 367
Working memory maintenance^#^					
Baseline	621 ± 331	576 ± 244	499 ± 267	517 ± 265	517 ± 267
Follow-up	347 ± 173	276 ± 174	257 ± 149	255 ± 146	252 ± 145
Cognitive flexibility^#^					
Baseline	637 ± 241	645 ± 210	644 ± 250	622 ± 236	621 ± 236
Follow-up	374 ± 143	356 ± 126	377 ± 161	348 ± 146	341 ± 133
Response inhibition^#^					
Baseline	214 ± 173	235 ± 190	251 ± 191	248 ± 183	245 ± 185
Follow-up	245 ± 207	179 ± 189	210 ± 171	201 ± 164	202 ± 163

*A+D ADHD with onset of depression, *A* only ADHD, *D* only depression, *C* comparison; neither ADHD nor depression, *H* healthy; no psychiatric diagnoses

**in years; #Reaction time in milliseconds

Linear mixed model analyses with sex, age, and ADHD and depression medication use as covariates revealed significant group differences in response time variability at baseline: *F*(3,1517) = 3.92, *p* = .008, ɳ^2^ = 0.004; working memory maintenance at follow up: *F*(3,1515) = 2.56, *p* = .05, ɳ^2^ = 0.004, and; change in response time variability scores between baseline and follow-up: *F*(3,1515) = 3.35, *p* = .01, ɳ^2^ = 0.005. Baseline response time variability RT was higher in group A than group C (*p* = .001). Working memory maintenance RT at follow-up was higher in group A+D than group C (*p* = .007). Between baseline and follow-up, the slope of change in response time variability RT for group A was significantly more negative (*p* = .002) than that of group C. The covariates-corrected effect sizes for these three differences were *d* = 0.44, *d* = 0.60, and *d* = −0.47, respectively. Finally, results of post-hoc pairwise comparisons are presented in Table 2.

**Table 2:** Posthoc pairwise comparisons of groups for differences in reactions times for response time variability and working memory maintenance

Groups compared^*^	Response time variability-baseline			Working memory maintenance-follow-up		
	Mean difference	SE	p^#^	Mean difference	SE	p^#^
A+D v/s A	−123.74	147.66	0.40	64.38	50.19	0.20
A+D v/s D	134.99	121.22	0.26	97.79	41.28	0.01
A+D v/s C	151.50	117.95	0.19	95.97	40.16	0.01
A v/s D	258.74	96.71	0.008	33.40	32.94	0.31
A v/s C	275.25	92.45	0.003	31.59	31.47	0.31
D v/s C	16.51	35.78	0.64	−1.81	12.22	0.88

^*^ Groups: *A*+*D* ADHD with depression, *A* only ADHD, *D* only depression, *C* comparison

^#^ *p* values adjusted for multiple testing using the Benjamini-Hochberg False Discovery Rate

**Fig. 2:**
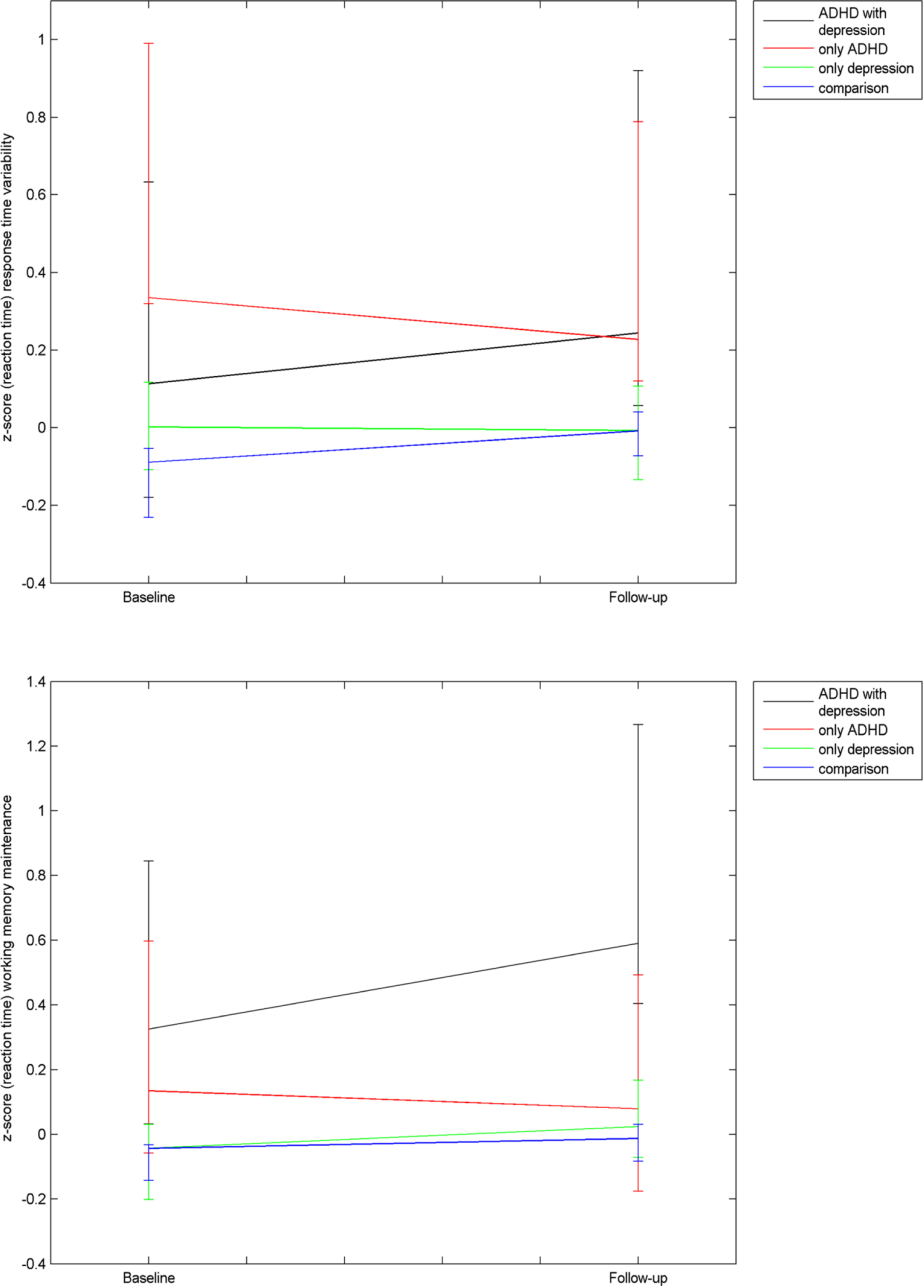
Reaction times for working memory maintenance and response time variability at baseline and follow-up

Figure 2 presents RTs of the four groups for response time variability and working memory maintenance functions at the 2 time-points. Linear mixed model analyses on these RT scores (without including covariates) yielded similar group differences in response time variability at baseline: *F*(3,1521) = 4.23, *p* = .005, ɳ^2^ = 0.003; working memory maintenance at follow up: *F*(3,1521) = 2.56, *p* = .05, ɳ^2^ = 0.002, and; change in response time variability scores between baseline and follow-up: *F*(3,1516) = 3.36, *p* = .01, ɳ^2^ = 0.004 and likewise indicated a higher baseline response time variability RT in group A than group C (*p* = .001); higher working memory maintenance RT at follow-up in group A+D than group C (*p* = .007); and a more negative slope of change in response time variability RT for group A relative to group C (*p* = .002). The effect sizes for these three differences were *d* = 0.52, *d* = 0.60, and *d* = −0.55, respectively.

For the first sensitivity analysis, participants from group C with psychiatric diagnoses (other than ADHD or depression) were excluded (separation anxiety: *n* = 19; agoraphobia: *n* = 6; conduct disorder: *n* = 71; generalised anxiety disorder: *n* = 22; oppositional defiant disorder: *n* = 65; panic disorder: *n* = 13; separation anxiety disorder: *n* = 25; social phobia: *n* = 110; specific phobia: *n* = 110, total *n* = 323) to form a new group of 888 adolescents (group H; healthy). Linear mixed effects analyses revealed group differences in baseline response time variability: *F*(3,1207) = 4.64, *p* = .003, ɳ^2^ = 0.007; change in response time variability between baseline and follow-up: *F*(3,1200) = 3.99, *p* = .008, ɳ^2^ = 0.005, and; follow-up working memory maintenance: *F*(3,1198) = 2.76, *p* = .041, ɳ^2^ = 0.005. Results of group and posthoc pairwise comparisons for response time variability and working memory maintenance were comparable to results from the main analysis. In addition, group differences in baseline processing speed were found: *F*(3,1201) = 2.90, *p* = .03, ɳ^2^ = 0.006. None of the group differences in processing speed remained significant after applying FDR corrections.

For the second sensitivity analysis, 158 participants with a CBCL or TCP ADHD score with *SD* > 1.5 were excluded from the comparison group to form a new comparison group of 1391 adolescents. Linear mixed effects analyses revealed group differences in baseline response time variability: *F*(3, 1373) = 7.05, *p* < .001, ɳ^2^ = 0.007; follow-up working memory maintenance: *F*(3, 1358) = 3.62, *p* = .013, ɳ^2^ = 0.005, and; change in response time variability between baseline and follow-up: *F*(3, 1369) = 5.42, *p* = .001, ɳ^2^ = 0.006. Results of group and posthoc pairwise comparisons for response time variability and working memory maintenance were comparable to the results from the main analysis. Additionally, group differences were found in baseline processing speed: *F*(3, 1353) = 3.13, *p* = .025, ɳ^2^ = 0.006). Post hoc pairwise comparisons showed that group A was significantly slower (FDR corrected) than group C (mean difference = 12.78, *SE* = 4.63, *p* = .006).

## Discussion

This study was aimed at assessing if ADHD with an onset of depression is associated with a unique cognitive functioning profile in adolescents with retrospectively self-reported ADHD and depression. Results show that cognitive functioning in adolescents with ADHD plus depression and only ADHD differed from the control groups either with or without depression. In particular, working memory maintenance of adolescents with ADHD plus depression at mean age 19 years was poor as compared to healthy adolescents and adolescents with only depression. Furthermore, the group of adolescents with only ADHD performed worse in response time variability at mean age 11 than the depressed and comparison groups. Our results also suggest that response time variability function improved between early adolescence and young adulthood in adolescents with only ADHD. We found no evidence, however, for cognitive functioning differences between adolescents with ADHD who did and did not develop depression.

The presence of comorbidities in individuals with ADHD is believed to increase the severity of cognitive deficits (Crawford et al. 2006). Consequently, we suspected that adolescents with ADHD and comorbid depression may have a poorer cognitive functioning than adolescents with only ADHD. We found no differences specifically between these two groups on our measures. Further, based on previous research, we suspected that an onset of depression in adolescents with ADHD may be preceded by impaired cognitive functioning (Austin et al. 2001). Our results did not support this either; adolescents with ADHD who developed depression did not differ from healthy comparisons at the baseline assessments. We also suspected that the development of depression would interfere with the process of cognitive maturation in adolescents with ADHD. Results show that adolescents with only ADHD showed an improvement in their cognitive (response time variability) functioning between ages of 11 and 19 years. Adolescents with ADHD who developed additional depression, did not show any cognitive improvements.

Adolescents with ADHD plus an onset of depression showed poorer working memory maintenance than comparison adolescents at a mean age of 19 years. The presence of memory maintenance difficulties at late adolescence in our sample may be related to the development of comorbid depression in adolescents with ADHD. Based on self-reported symptoms, we found that these adolescents showed increasing depressive symptoms for at least 6 years between the second and fourth assessment waves. ADHD symptom scores, on the other hand, did not change much for this comorbid group. It is possible that the upcoming depression and not ADHD played a role in the poor working memory maintenance performance at late adolescence. However, adolescents with only depression had a similarly increasing depressive symptom profile but did not show poor cognitive functioning in any domain (possible reasons for which we discuss later). It can be speculated that the development of depression may have increased the cognitive burden associated with ADHD and led to working memory maintenance difficulties. Alternately, poor cognitive functioning may have led to the development of depression in adolescents with ADHD.

Working memory maintenance, at age 11, of adolescents with ADHD and either with or without depression was comparable to that of the comparison group. Previous studies though, show impaired working memory in children with ADHD at an early age. Three related reasons as to why we did not find any group differences in working memory maintenance at age 11 in this study are worth mentioning. First, previous studies have been mostly based on recall tasks, which unlike recognition based tasks, such as the memory search task of the ANT, are more likely to show differences between children with and without ADHD early on (Rapport et al. 2000). Second, working memory maintenance assessed by visual array tasks, as in the ANT, is the total information held in working memory at any given point (Rapport et al. 2013; Shipstead et al. 2012). This component of working memory has been shown to be only minimally affected in children with ADHD (Raiker et al. 2012). In contrast, the central executive components of working memory, as assessed by complex span tasks, differ from working memory maintenance by being involved in the active processing of information held internally and are not related to the storage or maintenance of information. The central executive components of working memory are more often impaired than working memory maintenance in children with ADHD (Kasper et al. 2012; Rapport et al. 2013; Shipstead et al. 2012). Third, it is possible that the memory search task of the ANT measures short-term memory (Cowan 2008), which is likewise less impaired in children with ADHD (Dovis et al. 2015).

Adolescents with only ADHD showed poorer response time variability than comparison adolescents. Response time variability performance, however, improved with time in the group with only ADHD. For this group, self-reported ADHD symptoms also showed a decrease between early and late adolescence. It is likely that a decline in ADHD symptom severity was related to the improving response time variability performance.

Amongst adolescents with ADHD, results showed that the response time variability domain was affected at early adolescence while working memory maintenance was affected at young adulthood. The maturation of various cognitive domains occurs at different ages (Anderson 2002; Anderson et al. 2001; Boelema et al. 2013; Huizinga et al. 2006; Luna and Sweeney 2004), which may explain why response time variability was affected first followed by working memory maintenance later and not the other way around. The maturation of working memory peaks late in adolescence (Boelema et al. 2013; Huizinga et al. 2006; Luna and Sweeney 2004) and response time variability may therefore be affected prior to working memory maintenance.

Previous studies have reported cognitive flexibility, focused attention, and response inhibition impairments in individuals with ADHD (Nigg 2005). We did not find deficits in these three cognitive domains amongst adolescents with ADHD. One explanation for this could relate to the age of assessments in our sample (van Lieshout et al. 2013). The vast majority of research on ADHD-related cognitive deficits involves younger children, while we included adolescent participants. Further, core cognitive deficits proposed to be etiologically related to ADHD are less evidently present in adolescence (despite the presence of ADHD), suggesting that some children have sufficient cognitive maturation and do not show deficits (Thissen et al. 2014). Moreover, various cognitive domains mature at different ages (Anderson 2002; Anderson et al. 2001; Boelema et al. 2013; Luna and Sweeney 2004). It is thus, possible that several cognitive functions had matured sufficiently in our sample by the first assessment time point and therefore did not show group differences.

Some studies have suggested that only response inhibition deficits persist in the longer term while other cognitive domains mature sufficiently irrespective of remission from ADHD (Lei et al. 2015; McAuley et al. 2014). In this respect, our results may be remarkable in showing a lack of response inhibition deficits at young adulthood. However, these studies were based on patient populations of ADHD with severe problems and often multiple comorbidities. Our research findings are based on ADHD cases selected from the general population, who may not show severe cognitive functioning problems. Another alternative explanation comes from recent studies suggesting that ADHD-related cognitive deficits may be seen only for domains of working memory and sustained attention (Rapport et al. 2013), but not response inhibition (Alderson et al. 2007; Lijffijt et al. 2005). A final explanation for not finding response inhibition deficits, as in many previous studies that reported no deficits, may be related to the task differences among studies. Withholding a response on some trials (such as in the Stop task and Go/NoGo task) and withholding a compatible and automatic response on all trials (as in the ANT) may tap into different cognitive processes (Rommelse et al. 2007). Overall, the association of response inhibition deficits with ADHD is widely debated and further research may be needed to fully understand the heterogeneity in current literature.

A recent review of the literature has revealed that several domains of cognitive functioning including flexibility, attention, speed, and inhibition are affected in individuals with depression (Snyder 2013). Our results did not show impaired functioning in any domain for adolescents with only depression. The majority of studies on cognitive dysfunction in depressed individuals are limited to adulthood or even late-adulthood (Han et al. 2012). Cognitive dysfunction in adolescents with depression may not necessarily be similar to that in adults. It is also likely that depression-related cognitive deficits are not present pre-morbidly or shortly after onset, but develop only gradually if the depression persists or re-occurs over time. In this case, these deficits may not have fully emerged in our sample of young adults with relatively recently developed depressive problems.

An important limitation of our study concerns the use of the CIDI to establish diagnoses of ADHD. Based on parent and teacher reports available at baseline, it was found that ADHD symptom scores were low in the CIDI diagnosed ADHD groups: less than half the participants in the two ADHD groups scored greater than 1.5 standard deviations on either the parent or teacher ratings. The CIDI is a well-validated interview (Wittchen 1994), but the ADHD section is of a relatively recent date, and has therefore been used in only a limited number of studies so far (de Graaf et al. 2008; Kessler et al. 2006, 2010; Lara et al. 2009). The CIDI assesses the presence of self-reported symptoms during a structured interview which are used to construct lifetime as well as current diagnoses according to the DSM-IV. The CIDI- based ADHD diagnosis includes the presence of functioning impairments. Given the age of the sample at the time of the diagnostic interview (about 19 years) and the availability of fully standardized diagnostic interviews at this age, we used the best possible interview to determine the presence or absence of ADHD. Furthermore, the prevalence rates derived from the CIDI are comparable to that obtained from other population-based studies in adulthood (Merikangas et al. 2010). Although previous studies have used information form the respondent to establish ADHD diagnoses in adulthood (de Graaf et al. 2008; Kessler et al. 2006; Lara et al. 2009), in childhood this practice is uncommon. The use of the respondent-only assessments as opposed to multiple-informant based assessments, may underestimate the actual prevalence of ADHD or misdiagnose individuals as having ADHD (Sibley et al. 2012), and this likely also holds for adults (Privitera et al. 2015). Young adults, like children and adolescents, may not have a full appreciation of their symptoms or impairments and in our sample we may have found additional individuals with a diagnosis of ADHD had we also conducted a parent-interview. Currently, those individuals with (possible) parent but not self-recognized ADHD are part of the no problem group and this misclassification may have led to finding less outspoken cognitive differences between the groups. However, additional post-hoc analyses indicated that removing participants with a high parent/teacher ADHD score at baseline and without a CIDI ADHD diagnosis yielded highly similar findings. One exception was that children with ADHD only were slower on baseline speed at age 11. We conclude that current findings pertain to participants diagnosed with ADHD who themselves recognize their childhood onset ADHD symptoms and impairments. We have nonetheless no reason to doubt the validity of these findings since removal of possible false negatives (i.e. cases we would have identified through a parent interview) did not alter the conclusions of our study.

Other limitations to the manuscript include the following: first, the number of adolescents with ADHD only and ADHD combined with depression was low in our sample. Because of this, some group differences may not have reached statistical significance. Conversely, some group differences may have reached significance at smaller effect sizes. Second, as the incidence of depression remains high after 19 years of age, a part of the group with only ADHD (as well as part of the control group) may still develop depression later on. This may have led to an incorrect grouping of adolescents, and possible under-estimation of effects. Third, we did not withdraw participants from stimulant medication prior to cognitive testing although this did not seem to influence our findings.

Thus far, it has been difficult to pinpoint the exact cognitive profile associated with ADHD with affected cognitive domains varying widely across studies. Cognitive function is assumed to change throughout development in patients with ADHD and to generally improve with time (Coghill et al. 2014; Halperin and Schulz 2006; Rajendran et al. 2013). These age-dependent changes may explain some of the discrepancies found in studies. Apart from age-related cognitive deficits, the presence of comorbidities may also explain the heterogeneity of cognitive profiles associated with ADHD. Previously, studies have shown that comorbid anxiety and oppositional defiant disorder can markedly change the cognitive profiles of children and adolescents with ADHD (Rhodes et al. 2012; Vance et al. 2013). Our results also show cognitive differences amongst adolescents with and without comorbid depression, and further reveal that these profiles vary with age. Future studies may benefit from including assessments of comorbidities and age-dependent changes to better capture the nature of cognitive functioning associated with ADHD.

## Electronic supplementary material

Below is the link to the electronic supplementary material.

ESM 1 (PDF 96 kb)

ESM 2 (DOC 116 kb)

ESM 3 (DOCX 42 kb)
