# Supplementary material for: Cognitive Functioning in Adolescents with Self-Reported ADHD and Depression: Results from a Population-Based Study
Source: J Abnorm Child Psychol. 2016 May 3;45(1):69–81. doi: 10.1007/s10802-016-0160-x (PMC5219031; doi:10.1007/s10802-016-0160-x)
Supplement: Supplementary file 1 — (PDF 96 kb) [file 10802_2016_160_MOESM1_ESM.pdf]

S1. Supplementary material: Overview of the mean age and gender distributions of participants in the TRAILS sample

[Cognitive functioning in adolescents with self-reported ADHD and depression: results from a population-based study]

Authors: Arunima Roy, MBBS, Albertine J. Oldehinkel, PhD, Catharina A. Hartman, PhD

Interdisciplinary Centre Psychopathology and Emotion regulation, University of Groningen, University Medical Centre Groningen, The Netherlands

Address correspondence to: Arunima Roy, Interdisciplinary Centre Psychopathology and Emotion regulation (ICPE), University Medical Centre Groningen, CC 72, P.O. Box 30.001, 9700 RB Groningen, the Netherlands. F + 31 50 361 9722, e-mail: r.roy@umcg.nl

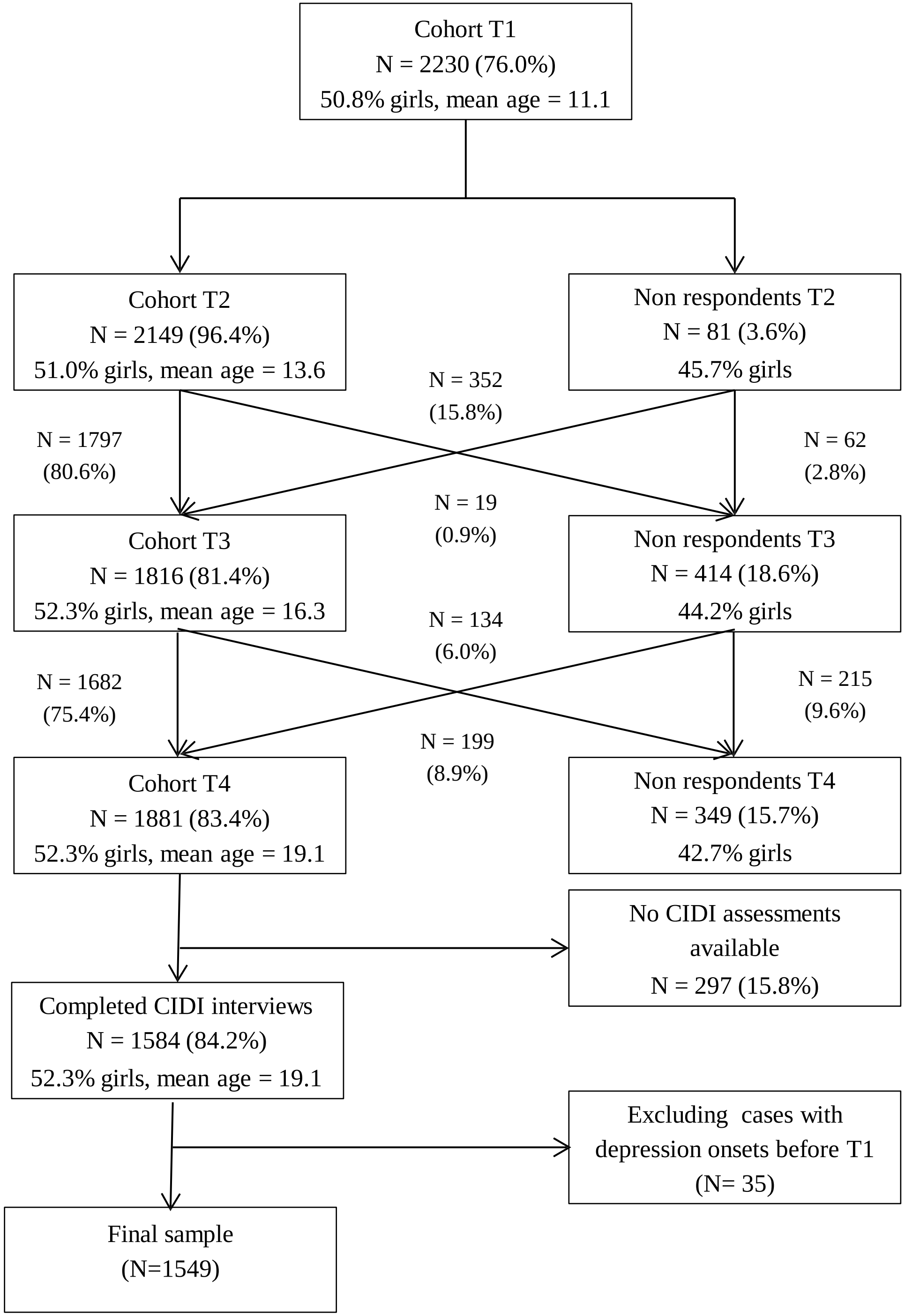
Cohort T1
N = 2230 (76.0%)
50.8% girls, mean age = 11.1
Cohort T2
N = 2149 (96.4%)
51.0% girls, mean age = 13.6
Non respondents T2
N = 81 (3.6%)
45.7% girls
N = 352
(15.8%)
N = 1797
(80.6%)
N = 62
(2.8%)
N = 19
(0.9%)
Cohort T3
N = 1816 (81.4%)
52.3% girls, mean age = 16.3
Non respondents T3
N = 414 (18.6%)
44.2% girls
N = 134
(6.0%)
N = 1682
(75.4%)
N = 215
(9.6%)
N = 199
(8.9%)
Cohort T4
N = 1881 (83.4%)
52.3% girls, mean age = 19.1
Non respondents T4
N = 349 (15.7%)
42.7% girls
No CIDI assessments
available
N = 297 (15.8%)
Completed CIDI interviews
N = 1584 (84.2%)
52.3% girls, mean age = 19.1
Excluding cases with
depression onsets before T1
(N= 35)
Final sample
(N=1549)
