# Supplementary material for: Cognitive Functioning in Adolescents with Self-Reported ADHD and Depression: Results from a Population-Based Study
Source: J Abnorm Child Psychol. 2016 May 3;45(1):69–81. doi: 10.1007/s10802-016-0160-x (PMC5219031; doi:10.1007/s10802-016-0160-x)
Supplement: Supplementary file 2 — (DOC 116 kb) [file 10802_2016_160_MOESM2_ESM.doc]

**S2. Supplementary Material:** The Amsterdam Neuropsychological Tasks (ANT): task descriptions

[Cognitive functioning in adolescents with self-reported ADHD and depression: results from a population-based study]

**Authors:** Arunima Roy, MBBS, Albertine J. Oldehinkel, PhD, Catharina A. Hartman, PhD

Interdisciplinary Centre Psychopathology and Emotion regulation, University of Groningen, University Medical Centre Groningen, The Netherlands

**Address correspondence to:** Arunima Roy, Interdisciplinary Centre Psychopathology and Emotion regulation (ICPE), University Medical Centre Groningen, CC 72, P.O. Box 30.001, 9700 RB Groningen, the Netherlands. F + 31 50 361 9722, e-mail: [r.roy@umcg.nl](mailto:r.roy@umcg.nl)

The Amsterdam Neuropsychological Tasks (ANT): task descriptions

The Amsterdam Neuropsychological Tasks program (ANT) (De Sonneville 1999) is a computer aided assessment battery of response time tasks that allow for the systematic evaluation of cognitive capacities. The ANT has proven to be a sensitive and valid tool in non-referred (De Sonnevil*le et* al 2002, Gro*ot et* al 2004, Sti*ns et* al 2005) as well as referred samples of various clinical domains such as minor neurological dysfunction (de Sonnevil*le et* al 1993), attention deficit disorders (Hanis*ch et* al 2004, Slaats-Willem*se et* al 2003), and autism-related disorders (Altha*us et* al 1996). For the present study, five subtasks from the ANT were selected and these are further described below:

1. Baseline speed task

A white fixation cross is projected at the centre of the screen that changes into a white square at random time intervals (Fig. 1). Children are instructed to press a mouse button with their index finger as soon as the white cross is seen. The task consists of two parts, each with 10 practice trials and 32 test trials. The first part requires responses from the non-dominant hand and the second part responses from the dominant hand. The valid response window (VRW) ranges from 150-4000 milliseconds (ms). The post response interval (PRI) ranges from 500 to 2500 ms. The measure ‘processing speed’ is calculated as the mean reaction time (RT) over both the non-dominant and dominant hand responses.


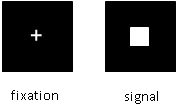


Fig. 1

1. Pattern recognition task

Children are instructed to memorize a predefined target pattern following which they are asked to recognize this pattern from a display set containing four patterns. Patterns contained in half of the display sets appear dissimilar to the target and the other half contain patterns appearing similar to the target (Fig. 2). The task consists of 12 practice trials and 80 test trials. Of the 80 test trails, half require responses with the non-dominant hand and the other half require responses with the dominant hand. The VRW is 200-7000 ms and the PRI is 1200 ms. The measure ‘focussed attention’ is computed by subtracting the mean RT of the dissimilar trials from the similar trials.


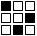


(Target pattern)


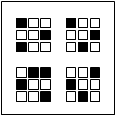

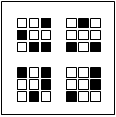

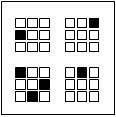

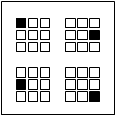


(Target similar) (Non-target similar) (Target dissimilar) (Non-target

dissimilar)

Fig. 2

1. Sustained attention task

Six hundred dot patterns are successively presented on the screen in 50 series of 12 trials. Each series consists of four 3-, 4-, and 5-dot patterns, presented in a pseudo random sequence. Participants responded by pressing either the left or the right mouse button, depending on the stimulus. Responses to 4-dot patterns were made by pressing the mouse button with the dominant hand (‘yes response’) (Fig. 3). Responses to 3-, or 5-dot patterns were made by pressing the mouse button with the non-dominant hand (‘no response’). Task assessments are preceded by 24 practice trials. The measure ‘response time variability’ is computed as the within-subject standard deviation (i.e. the variability) of the mean RT of the 50 series and is interpretable as a measure of response stability in continuous task performance.


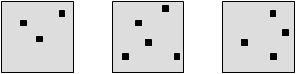


Non target Non target Target

Fig. 3

1. Memory search task

Children are instructed to memorise one (part 1, 40 trials), two (part 2, 72 trials), and three (part 3, 96 trials) target consonant(s). Display sets are subsequently presented that consist of four consonants (Fig. 4). Display sets in half the trials contain the complete target set requiring a ‘yes-response’ (pressing mouse button by the dominant hand). In the other half, display sets contain none of the target letters or an incomplete target set, requiring a ‘no-response’ (pressing mouse button by non-dominant hand). Each task (part) is preceded by 12 practice trials. The VRW is 200-8000 ms and the PRI 1200 ms. The measure ‘working memory maintenance’ is computed by subtracting the mean RT of part 1 low working memory load) target trials from the mean RT of part 3 (high working memory maintenance load) target trials.

Target Non-Target Non-Target Non-Target

0 distractors 1 distractor 2 distractors

r

v

k

s

j

f

z

d

c

r

b

m

s

h

t

k

Fig. 4

1. Shifting set task

A horizontal bar is presented in the centre of the screen. In each trial, a coloured square moves across the bar randomly, to either to the left or to right (fig 5). The task consists of three parts. In part 1, children are instructed to copy the movement of a green coloured square (i.e. a green square moving to the left requires pressing the left mouse button, and a green square moving to the right requires pressing the right mouse button). In part 2, children are instructed to ‘mirror’ the direction of a red coloured square (i.e. a leftward moving red square requires pressing the right mouse button, and a rightward moving red button requires pressing the left mouse button). In part 3, the colour of the moving square randomly alternates between green and red. When the square is green, children are required to ‘copy’ its movement (i.e. responses as in part 1), and when the square is red, children are required to ‘mirror’ its movement (i.e. responses as in part 2). Parts 1 and 2 consist of 40 test trials each and are each preceded by 10 practice trials. Part 3 consists of 80 test trials, preceded by 16 practice trials. The VRW is 150 – 6000 ms and the PRI 250 ms. The measure ‘response inhibition’ is computed by subtracting the mean RT of part 1 from the mean RT of part 2. The measure ‘cognitive flexibility’ is computed by subtracting the mean RT of part 1 from the mean RT of part 3.

(
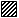
= red,
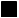
 = green)


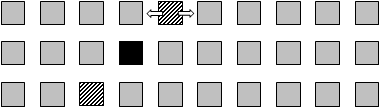


Trial 1

Trial 2

Trial 3

Fig. 5

**References:**

Althaus M, De Sonneville LM, Minderaa RB, Hensen LG, Til RB (1996). Information processing and aspects of visual attention in children with the DSM-III-R diagnosis “Pervasive developmental disorder not otherwise specified”(PDDNOS): I. Focused and divided attention*. Child Neuropsychology* 2, 17-29

de Sonneville LM, Geeraets MH, Woestenburg JC (1993). Information processing in children with minor neurological dysfunction: behavioural and neurophysiological indices*. Early human development* 34, 69-78

De Sonneville L (1999). Amsterdam Neuropsychological Tasks: A computer-aided assessment program*. Computers in psychology* 6, 187-203

De Sonneville L, Verschoor C, Njiokiktjien C, Op het Veld V, Toorenaar N, Vranken M (2002). Facial identity and facial emotions: Speed, accuracy, and processing strategies in children and adults*. Journal of Clinical and Experimental Neuropsychology* 24, 200-213

Groot AS, De Sonneville LM, Stins JF, Boomsma DI (2004). Familial influences on sustained attention and inhibition in preschoolers*. Journal of Child Psychology and Psychiatry* 45, 306-314

Hanisch C, Konrad K, Günther T, Herpertz-Dahlmann B (2004). Age-dependent neuropsychological deficits and effects of methylphenidate in children with attention-deficit/hyperactivity disorder: a comparison of pre-and grade-school children*. Journal of neural transmission* 111, 865-881

Slaats-Willemse D, Swaab-Barneveld H, De Sonneville L, Van Der Meulen E, Buitelaar J (2003). Deficient response inhibition as a cognitive endophenotype of ADHD*. Journal of the American Academy of Child & Adolescent Psychiatry* 42, 1242-1248

Stins J, De Sonneville LM, Groot AS, Polderman TC, Van Baal CG, Boomsma DI (2005). Heritability of selective attention and working memory in preschoolers*. Behavior genetics* 35, 407-416
