# Supplementary material for: Cognitive Functioning in Adolescents with Self-Reported ADHD and Depression: Results from a Population-Based Study
Source: J Abnorm Child Psychol. 2016 May 3;45(1):69–81. doi: 10.1007/s10802-016-0160-x (PMC5219031; doi:10.1007/s10802-016-0160-x)
Supplement: Supplementary file 3 — (DOCX 42 kb) [file 10802_2016_160_MOESM3_ESM.docx]

**S3. Supplementary Material:** Information on medication use and validity of CIDI diagnoses

[Cognitive functioning in adolescents with self-reported ADHD and depression: results from a population-based study]

**Authors:** Arunima Roy, MBBS, Albertine J. Oldehinkel, PhD, Catharina A. Hartman, PhD

Interdisciplinary Centre Psychopathology and Emotion regulation, University of Groningen, University Medical Centre Groningen, The Netherlands

**Address correspondence to:** Arunima Roy, Interdisciplinary Centre Psychopathology and Emotion regulation (ICPE), University Medical Centre Groningen, CC 72, P.O. Box 30.001, 9700 RB Groningen, the Netherlands. F + 31 50 361 9722, e-mail: [r.roy@umcg.nl](mailto:r.roy@umcg.nl)

Table I Percentage of participants in each group receiving medications for ADHD or depression at the four time points

| **Groups** | Dexamphetamine | | | | Methylphenidate | | | |
| --- | --- | --- | --- | --- | --- | --- | --- | --- |
|  | T1 | T2 | T3 | T4 | T1 | T2 | T3 | T4 |
| A + D | 0 | 0 | 0 | 4.7 | 0 | 4.7 | 0 | 38.1 |
| A | 0 | 0 | 0 | 2.8 | 5.7 | 0 | 8.6 | 20.0 |
| D | 0 | 0 | 0 | 0 | 0 | 8.5 | 1.1 | 2.1 |
| C | 0 | 0.2 | 0.1 | 0 | 0.4 | 5.2 | 0.2 | 1.5 |
|  | Atomoxetine | | | | Venlafaxine | | | |
|  | T1 | T2 | T3 | T4 | T1 | T2 | T3 | T4 |
| A + D | 0 | 0 | 0 | 0 | 0 | 0 | 0 | 0 |
| A | 0 | 5.7 | 0 | 0 | 0 | 0 | 0 | 0 |
| D | 0 | 0 | 0 | 0 | 0 | 0 | 0 | 0.7 |
| C | 0 | 0 | 0 | 0 | 0 | 0 | 0 | 0 |
|  | Imipramine | | | | Clomipramine | | | |
|  | T1 | T2 | T3 | T4 | T1 | T2 | T3 | T4 |
| A + D | 0 | 0 | 0 | 0 | 0 | 0 | 0 | 0 |
| A | 0 | 0 | 0 | 0 | 0 | 0 | 0 | 0 |
| D | 0 | 0 | 0 | 0 | 0 | 0.7 | 0 | 0 |
| C | 0.1 | 0 | 0 | 0 | 0 | 0.1 | 0 | 0 |
|  | Amitryptyline | | | | Nortriptyline | | | |
|  | T1 | T2 | T3 | T4 | T1 | T2 | T3 | T4 |
| A + D | 0 | 0 | 0 | 0 | 0 | 0 | 0 | 0 |
| A | 0 | 0 | 0 | 0 | 0 | 0 | 0 | 0 |
| D | 0 | 0.7 | 0 | 0 | 0 | 0 | 0 | 0 |
| C | 0 | 0.1 | 0 | 0 | 0 | 0.2 | 0 | 0 |
|  | Fluoxetine | | | | Citalopram | | | |
|  | T1 | T2 | T3 | T4 | T1 | T2 | T3 | T4 |
| A + D | 0 | 0 | 0 | 0 | 0 | 0 | 0 | 0 |
| A | 0 | 0 | 0 | 0 | 2.8 | 0 | 0 | 0 |
| D | 0 | 0 | 0.3 | 1.1 | 0 | 0 | 0 | 1.7 |
| C | 0 | 0 | 0 | 0 | 0 | 0.1 | 0 | 0.1 |
|  | Paroxetine | | | | Sertraline | | | |
|  | T1 | T2 | T3 | T4 | T1 | T2 | T3 | T4 |
| A + D | 0 | 0 | 0 | 0 | 0 | 0 | 0 | 0 |
| A | 0 | 0 | 0 | 0 | 0 | 0 | 0 | 0 |
| D | 0 | 0 | 0.3 | 0.3 | 0 | 0 | 0 | 0 |
| C | 0 | 0.1 | 0 | 0 | 0 | 0 | 0 | 0.2 |
|  | Fluvoxetine | | | | Moclobemide | | | |
|  | T1 | T2 | T3 | T4 | T1 | T2 | T3 | T4 |
| A + D | 0 | 0 | 0 | 0 | 0 | 0 | 0 | 0 |
| A | 0 | 0 | 0 | 0 | 0 | 0 | 0 | 0 |
| D | 0 | 0 | 0.3 | 0 | 0 | 0.7 | 0 | 0 |
| C | 0 | 0.1 | 0 | 0 | 0 | 0.1 | 0 | 0 |

Groups: A+D (ADHD with an onset of depression); A (only ADHD); D (only an onset of depression); C (comparison)

*N =* 1549

Table II Cognitive functioning differences between participants receiving and not receiving medications

| Baseline | | | | | |
| --- | --- | --- | --- | --- | --- |
|  | Participants with medications  RT - Mean (SD) | Participants without medications  RT - Mean (SD) | t | df | p |
| Processing  speed | 289.1 (7.97) | 331.6 (38.8) | 1.89 | 330 | .06 |
| Focussed attention | 1463.6 (457.5) | 1473.6 (465.8) | .02 | 329 | .99 |
| Response time variability | 2420.0 (810.0) | 1747.0 (842.7) | -1.38 | 334 | .17 |
| Working memory maintenance | 699.4 (60.8) | 515.98 (274.6) | -1.21 | 332 | .29 |
| Response inhibition | 178.3 (237.9) | 249.6 (194.2) | .63 | 332 | .53 |
| Cognitive flexibility | 572.3 (115.6) | 648.7 (251.0) | .52 | 326 | .61 |
| Follow-up | | | | | |
|  | Participants with medications  RT - Mean (SD) | Participants without medications  RT - Mean (SD) | t | df | p |
| Processing  speed | 252.8 (26.5) | 252.0 (24.2) | -.19 | 327 | .85 |
| Focussed attention | 840.4 (310.8) | 807.8 (259.0) | -.65 | 331 | .51 |
| Response time variability | 877.8 (357.3) | 880.3 (382.1) | .04 | 333 | .97 |
| Working memory maintenance | 313.6 (183.6) | 807.8 (259.0) | -1.79 | 335 | .07 |
| Response inhibition | 226.8 (220.0) | 207.4 (169.6) | -1.01 | 324 | .31 |
| Cognitive flexibility | 403.0 (176.3) | 373.1 (155.0) | -.19 | 327 | .85 |

RT – Reaction time

Table III Standardized scores of self- (YSR and ASR), parent- (CBCL) and teacher-reported (TCP) ADHD symptoms in the four groups across the four assessment time-points

| **Assessment time points^*^** | **Groups^#^** | | | | **ANOVA** | |
| --- | --- | --- | --- | --- | --- | --- |
|  | **A + D**  mean (SD) | **A**  mean (SD) | **D**  mean (SD) | **C**  mean (SD) | **F** | **p** |
| **T1** |  |  |  |  |  |  |
| YSR | 0.45 (0.95) | 0.86 (1.11) | 0.16 (0.95) | -0.07 (0.99) | 14.78 | <.001 |
| CBCL | 0.78 (1.33) | 1.15 (1.02) | 0.06 (0.94) | -0.05 (0.98) | 19.85 | <.001 |
| TCP | 0.65 (1.25) | 0.39 (1.23) | -0.02 (0.98) | -0.01 (0.98) | 3.84 | .009 |
| **T2** |  |  |  |  |  |  |
| YSR | 0.95 (1.28) | 0.94 (1.06) | 0.16 (0.96) | -0.08 (0.97) | 22.03 | <.001 |
| CBCL | 0.99 (1.36) | 1.13 (1.03) | 0.23 (0.93) | -0.10 (0.96) | 26.96 | <.001 |
| TCP | 0.45 (1.17) | 0.92 (1.24) | 0.002 (0.96) | -0.03 (0.98) | 7.89 | <.001 |
| **T3** |  |  |  |  |  |  |
| YSR | 0.99 (1.36) | 1.13 (1.03) | 0.23 (0.93) | -0.10 (0.96) | 30.71 | <.001 |
| CBCL | 1.21 (1.51) | 1.49 (1.44) | 0.10 (0.90) | -0.08 (0.94) | 36.28 | <.001 |
| TCP | 0.60 (0.80) | 0.79 (1.19) | 0.09 (0.99) | -0.05 (0.98) | 6.46 | <.001 |
| **T4** |  |  |  |  |  |  |
| ASR | 1.98 (1.17) | 1.18 (1.24) | 0.43 (1.01) | -0.16 (0.89) | 78.90 | <.001 |

*ADHD symptoms were measured using Youth Self Reports (YSR), Child Behavior Checklists (CBCL) and Teacher’s Checklist of Pathology (TCP) at the first (T1), second (T2), and third (T3) assessment waves. At the fourth wave (T4) ADHD symptoms were assessed using the Adult Self Reports (ASR)

# A+D: ADHD with depression; A: only ADHD; D: only depression; C: comparisons

Table IV Standardized scores of self- (YSR and ASR) and parent-reported (CBCL) depressive symptoms in the four groups across the four assessment time-points

| **Assessment**  **time points^*^** | **Groups^#^** | | | | **ANOVA** | |
| --- | --- | --- | --- | --- | --- | --- |
|  | **A + D**  Mean (SD) | **A**  Mean (SD) | **D**  Mean (SD) | **C**  Mean (SD) | **F** | **p** |
| **T1** |  |  |  |  |  |  |
| YSR | 0.40 (0.94) | 0.43 (1.23) | 0.35 (1.10) | -0.10 (0.94) | 19.80 | <.001 |
| CBCL | 1.01 (1.30) | 0.58 (1.20) | 0.28 (1.14) | -0.09 (0.92) | 20.76 | <.001 |
| **T2** |  |  |  |  |  |  |
| YSR | 0.32 (0.97) | 0.27 (0.88) | 0.57 (1.25) | -0.14 (0.87) | 44.53 | <.001 |
| CBCL | 1.08 (1.42) | 0.29 (1.26) | 0.38 (1.18) | -0.11 (0.90) | 27.02 | <.001 |
| **T3** |  |  |  |  |  |  |
| YSR | 1.02 (1.53) | 0.17 (0.94) | 0.75 (1.24) | -0.19 (0.81) | 79.26 | <.001 |
| CBCL | 1.01 (1.43) | 0.56 (1.16) | 0.54 (1.34) | -0.14 (0.83) | 40.05 | <.001 |
| **T4** |  |  |  |  |  |  |
| ASR | 1.55 (1.20) | 0.37 (1.20) | 0.79 (1.17) | -0.22 (0.80) | 117.78 | <.001 |

*Symptoms of depression were measured using Youth Self Reports (YSR) and Child Behaviour Checklists (CBCL) at the first (T1), second (T2), and third (T3) assessment waves and Adult Self Reports (ASR) at the fourth wave (T4)

# A+D: ADHD with depression; A: only ADHD; D: only depression; C: comparisons
